# Non-capsular based immunization approaches to prevent *Streptococcus pneumoniae* infection

**DOI:** 10.3389/fcimb.2022.949469

**Published:** 2022-09-26

**Authors:** Pedro H. Silva, Yaneisi Vázquez, Camilo Campusano, Angello Retamal-Díaz, Margarita K. Lay, Christian A. Muñoz, Pablo A. González, Alexis M. Kalergis, Susan M. Bueno

**Affiliations:** ^1^ Millennium Institute on Immunology and Immunotherapy, Santiago, Chile; ^2^ Departamento de Genética Molecular y Microbiología, Facultad de Ciencias Biológicas, Pontificia Universidad Católica de Chile, Santiago, Chile; ^3^ Departamento de Biotecnología, Facultad de Ciencias del Mar y Recursos Biológicos, Universidad de Antofagasta, Antofagasta, Chile; ^4^ Unidad de Microbiología, Facultad de Ciencias de la Salud, Universidad de Antofagasta, Antofagasta, Chile

**Keywords:** Universal vaccines, *S. pneumoniae*, pneumonia, bacterial infections, immunity

## Abstract

*Streptococcus pneumoniae* is a Gram-positive bacterium and the leading cause of bacterial pneumonia in children and the elderly worldwide. Currently, two types of licensed vaccines are available to prevent the disease caused by this pathogen: the 23-valent pneumococcal polysaccharide-based vaccine and the 7-, 10, 13, 15 and 20-valent pneumococcal conjugate vaccine. However, these vaccines, composed of the principal capsular polysaccharide of leading serotypes of this bacterium, have some problems, such as high production costs and serotype-dependent effectiveness. These drawbacks have stimulated research initiatives into non-capsular-based vaccines in search of a universal vaccine against *S. pneumoniae.* In the last decades, several research groups have been developing various new vaccines against this bacterium based on recombinant proteins, live attenuated bacterium, inactivated whole-cell vaccines, and other newer platforms. Here, we review and discuss the status of non-capsular vaccines against *S. pneumoniae* and the future of these alternatives in a post-pandemic scenario.

## Introduction

*Streptococcus pneumoniae* (*S. pneumoniae*) is an opportunistic pathogen that colonizes the human nasopharynx (Henriques-Normark and Tuomanen, 2013; Weiser et al., 2018a). *S. pneumoniae* is a highly heterogeneous bacterium with more than 100 serotypes differentiated by their polysaccharide capsule composition (Pimenta et al., 2021). *S. pneumoniae* is a clinically relevant bacterium as is the bacterial leading cause of respiratory infections such as pneumonia, meningitis, otitis media, and other diseases in children under five years old and the elderly in the community (O’Brien et al., 2009; Wahl et al., 2018). Between 27-65% of children and 10% of adults over 65 years old carry *S. pneumoniae* in their nasopharyngeal tract as a commensal bacterium (Weiser et al., 2018a). Asymptomatic colonization plays a crucial role in transmission, infection, and invasive disease progression (Simell et al., 2012; Weiser et al., 2018a).

Currently, there are 2 types of vaccines against *S. pneumoniae* based on capsule polysaccharides. These vaccines are widely used in the world although their effectiveness is variable and depend upon several factors, including the epidemiologic profile of circulating *S. pneumoniae* serotypes in each geographical region where the vaccine is implemented or the age of individuals that received the vaccine (Daniels et al., 2016). Additionally, serotype replacement of pneumococcal infections with serotypes not contained in the vaccines has increased after the introduction of immunization programs in several places (Ladhani et al., 2018; Ouldali et al., 2021). Even though current pneumococcal conjugate vaccines (PCVs) have decreased the disease rate and severity generated by some serotypes, *S. pneumoniae* is capable of continually remodeling its genome, evolving antimicrobial resistance and capsular switching (Brueggemann et al., 2007; Ladhani et al., 2018; Ouldali et al., 2021). Because of this, in 2017, the WHO reported that *S. pneumoniae* is one of the 12 priority families of bacteria that have increased antibiotic resistance to several clinically relevant drugs (as penicillin) and high rates of disease (Tacconelli et al., 2018; Weiser et al., 2018a). Given this situation, we reviewed different vaccination strategies not based on capsule polysaccharides, which could improve the prevention of pneumococcal infections. This review seeks to highlight the importance of the design of vaccines that are not based on capsular polysaccharides and discuss their advantages, altogether approaching some recent works on the development of new vaccines against this pathogen.

## *Streptococcus pneumoniae* pathogenesis and current vaccines

*S. pneumoniae* is a Gram-positive, extracellular, capsulated, and fastidious microorganism. This bacterium has a genome of 2.16 Mbps, encoding 2,236 open reading frames (Tettelin, H. et al., 2001). Its genome is highly variable due to its natural ability to take up DNA through natural transformation (Johnsborg and Håvarstein, 2009; Andam and Hanage, 2015) and currently, 101 capsular serotypes of *S. pneumoniae* have been identified (Ganaie et al., 2020; Pimenta et al., 2021).

Pneumococcal propagation mainly occurs by person-to-person contact with droplet secretions generated by carriers when they cough and sneeze, known as *flugge* droplets (Hoge et al., 1994). In general, *S. pneumoniae* transmission occurs through the upper respiratory tract (URT) when respiratory infections increase during winter months (Sharma-Chawla et al., 2016). The expression of virulence factors that are necessary to colonize and invade the URT mucosal surface occurs after propagation (Weiser et al., 2018b). The following virulence factors are the most relevant in this process: i) Capsule, which plays a crucial role during infection, in fact, a study found that all clinical *S. pneumoniae* isolates from patients were encapsulated and that the type of capsule is related with the virulence produced in mice (Briles et al., 1992). In addition, unencapsulated *S. pneumoniae* has a reduced capacity to colonize the nasopharyngeal region in mice (Nelson et al., 2007). Other studies have shown that capsule is related to inhibition of classic and alternative complement pathways in serum from human donors (Hyams et al., 2010); ii) Pneumolysin (Ply) is a protein that induces cell lysis through membrane pore generation in several cell types (Tilley et al., 2005). Ply can also inhibit the complement system and produce DNA damage in alveolar epithelial cells *in vitro* (Alcantara et al., 2001; Rai et al., 2016; Brooks and Mias, 2018); iii) Pneumococcal surface protein A (PspA), which also promotes epithelial surface attachment and protects the bacterium against the complement system (Ren et al., 2003); iv) Other choline-binding proteins that modify the cell surface, allowing *S. pneumoniae* to bind to host cells (Maestro and Sanz, 2016); and v) IgA protease, which breaks down IgA (Janoff et al., 2014). Furthermore, other studies have found several *S. pneumoniae* virulence factors that alter the early innate immune response and initiate an adaptive immune response (Nieto et al., 2013; Zafar et al., 2017). Some of the virulence factors above have been used to develop vaccines against *S. pneumoniae* and will be discussed below (see **Table 1**).

**Table 1:** Streptococcus pneumoniae antigens.

Antigen	Localization	Reference
PspA	Cell surface	(Lu et al., 2015)
PsaA	Cell surface	(Lu et al., 2015)
Pht family	Cell surface	(Khan and Pichichero, 2012; André et al., 2020; Malekan et al., 2020)
Ply	Cytosol	(Alexander et al., 1994; Thanawastien et al., 2021)
LytB	Cell surface	(Corsini et al., 2016)

Among the available therapies to counteract *S. pneumoniae*, vaccines have successfully reduced the mortality and severity of pneumococcal diseases and decreased nasal carriage in the population (Troeger et al., 2018). Current pneumococcal vaccines are based on the polysaccharides present in the capsule, which is the main virulence factor of *S. pneumoniae* (see **Table 2**) (Mitchell and Mitchell, 2010). Nowadays, there are 2 types of vaccines widely used to prevent the disease. The pneumococcal polysaccharide vaccine (PPSV) and the pneumococcal polysaccharides conjugate vaccine (PCV) (Berical et al., 2016). The 23 valent pneumococcal polysaccharide vaccine (PPSV-23) contains purified polysaccharide from *S. pneumoniae* serotypes (showed in **Table 2**). This vaccine elicits 60- 70% of protection against capsular serotypes contained in the formulation in adults (Fine et al., 1994; Daniels et al., 2016). Unfortunately, PPSV-23 does not generate a robust production of antibodies in children under two years old likely due to their immune system is still in development (Leinonen et al., 1986; O’Brien et al., 1996; Daniels et al., 2016).

**Table 2:** Capsular Polysaccharide-based vaccines.

Vaccine	Serotypes Contained in the Vaccine	Efficacy	Limitation	Reference
*PPSV 23 (polysaccharide)*	1, 2, 3, 4, 5, 6B, 7F, 8, 9N, 9V, 10A, 11A, 12F, 14, 15B, 17F, 18C, 19A, 19F, 20, 22F, 23F, and 33F	Coverage of 23 serotypes. Effectiveness against IPD. Minimizing the severity of pneumonia.	Poor immunogenicity and effectiveness against pneumococcal pneumonia prevention.	(Daniels et al., 2016; Kim et al., 2017)
*PCV 7 (conjugate)*	4, 6B, 9V, 14, 18C, 19F, and 23F	Reduced invasive disease. Reduced carriage. Protective herd effect.	Increase in 3 and 19A infections.	(Lee et al., 2014)
*PCV 10 (conjugate)*	1, 4 5, 6B, 7F, 9V, 14, 18C, 19F, 23F	Reduced invasive disease. Reduced carriage.	Increase in 3 and 19A infections.	(Pilishvili et al., 2010; Lee et al., 2014)
*PCV 13 (conjugate)*	1, 3, 4, 5, 6A, 6B, 7F, 9V, 14, 18C, 19A, 19F, and 23F	Higher immunogenicity than PPV23. Effectiveness against pneumonia. Effectiveness against IPD.	Coverage of 13 serotypes only. High cost Increase in 35B infections.	(Olarte et al., 2017; Matanock et al., 2019)
*PCV15* *(conjugate)*	1, 3, 4, 5, 6A, 6B, 7F, 9V, 14, 18C, 19A, 19F, 22F, 23F and 33F	High immunogenicity. Effectiveness against pneumonia. Effectiveness against IPD.	Coverage of 15 serotypes only. High cost	(McFetridge et al., 2015; Stacey et al., 2019)
*PCV 20* *(conjugate) (recently approved by FDA)*	1, 3, 4, 5, 6A, 6B, 7F, 9V, 14, 18C, 19A, 19F, 23F, 8, 10A, 11A, 12F, 15B, 22F and 33F	Substantial IgG production and functional bactericidal immune response.	Coverage of 20 serotypes. High-cost production.	(Thompson et al., 2019)

To elicit a protective immune response in children under two years old, pneumococcal vaccines with polysaccharide antigens were conjugated with carrier proteins, which are known as 7 valent-pneumococcal polysaccharides conjugate vaccine (PCV7), 10 valent-PCV (PCV10), 13 valent-PCV (PCV13), 15 valent-PCV (PCV15) and 20 valent-PCV (PCV-20). PCV7 and PCV10 have been shown to induce protection against acute otitis media (AOM) and pneumonia (Pilishvili et al., 2010; Lee et al., 2014) and PCV13 markedly decreased pneumococcal pneumonia incidence of serotypes 19A and 3 in children under two years old (Olarte et al., 2017; Ladhani et al., 2018; Matanock et al., 2019). However, other studies indicate that some serotypes maintained their prevalence despite being included in the vaccine (e.g., 1, 3, 14, and 19A), and pneumococcal meningitis incidence did not decrease after the incorporation of the vaccine (Olarte et al., 2017; Ladhani et al., 2018; de Miguel et al., 2021; Ouldali et al., 2021). On the other hand, it seems that the introduction of PCVs has affected the serotype distribution and prevalence in the countries where it was implemented accompanied by an increase in the presence of other serotypes not included in the vaccine (Okike et al., 2014; Løchen et al., 2020; de Miguel et al., 2021; Ouldali et al., 2021). For example in China some studies showed that 19F, 19A, 23F, 14, 6A and 6B serotypes are the most prevalent in its population (Zhao et al., 2019; Wang et al., 2020; Liang et al., 2021). Other study from Japan showed that among children the predominant serotypes were not included in the 13-valent PCV (PCV13) like 15A, 35B, 23A, 15B (Kawaguchiya et al., 2020) and several studies have showed the emerging of 22F and 33F serotypes in the risk population (Sempere et al., 2020; Golden et al., 2021). Recently the Food and Drug Administration (FDA) approved a 20-valent vaccine from Pfizer, called PREVNAR 20, for adults ages 18 or older (Thompson et al., 2019). This vaccine includes the serotypes from PCV13 plus 7 additional serotypes (8, 10A, 11A, 12F, 15B, 22F, 33F). Vaccinated participants presented increased levels of immunoglobulin (Ig) G concentration in serum for all serotypes included and high opsonization activity. Moreover, an immunogenicity and safety clinical trial was performed in adults ≥ 65 years of age in February 2020 with an enrollment of 875 participants, people who received PREVNAR 20 showed no severe adverse effects like fever, muscular pain, among others (NCT03835975). Also, inoculated participants presented high titer of opsonophagocytic activity 1 month after immunization (Cannon et al., 2021).

## Non-capsular vaccines alternatives

As explained previously, capsular polysaccharide-based vaccines have contibuted to a significant decrease in pneumococcal infections, mainly pneumonia and AOM (Pilishvili et al., 2010; Lee et al., 2014). However, no vaccine formulation prevents serotype exchange and production of PCV vaccines is expensive (Krishnamoorthy et al., 2019). This has prompted research on new alternatives, the so-called non-capsular polysaccharide-based vaccines whose main characteristic is its universal properties (Berical et al., 2016; Mcdaniel and Swiatlo, 2021; Sempere et al., 2021). These vaccines are based on other approaches, including protein-based vaccines, inactivated whole-cell, or attenuated vaccines, which allow the use of general antigens from the pathogen that theoretically could protect from all *S. pneumoniae* serotypes. Several alternatives of these kind of vaccine candidates to promote immunity against *S. pneumoniae* have been evaluated in preclinical and clinical studies, which are discussed below (see **Table 3**).

**Table 3:** Non-capsular candidate vaccines in clinical trials.

Vaccine	Type of vaccine	Results	Reference
PspA	Protein-based vaccine	IgG production. Safe in adults.	(Bologa et al., 2012)
PspA + PhtD	Protein-based vaccine	IgG production. Safe in adults.	(Bologa et al., 2012)
GSK 2189242A	Protein-based vaccine	IgG production; It does not affect *S. pneumoniae* carriage.	(Odutola et al., 2016)
PnuBioVax	Protein-based vaccine	IgG production, the antibodies can inhibit the hemolytic activity and elicit neutralization capacity.	(Entwisle et al., 2017)
SPWCV	Whole cell-based vaccine	Safe and immunogenic. Phase 2 clinical trial.	(Keech et al., 2020)
ASP3772	Pneumococcal proteins as carrier	Safe in adults aged 18 to 64 years old and older adults aged 65 to 85 years old. Antibodies production with opsonophagocytic activity. Specific Th17 cells and increased IL-17 production.	(Chichili et al., 2022)

### Protein-based candidate vaccines

Protein-based vaccines are classical approaches that have been used to prevent infections caused by several bacterial and viral pathogens. The use of immunogenic proteins is a favorable strategy to induce a robust host immune response. Additionally, these proteins also should be well conserved among *S. pneumoniae* serotypes.

#### Preclinical studies

In general, virulence factors are immunogenic and well conserved targets to develop vaccines (Mitchell and Mitchell, 2010). Some examples are PsaA and PspA, which are important surface proteins that allow the attachment of *S. pneumoniae* to the epithelial cells and have been described that intranasal inoculation of these proteins alone or mixed elicit protection against nasopharyngeal carriage of *S. pneumoniae* in mice (Briles et al., 2000). In other study, immunization with a fusion protein that contain fragments from Pspa and PsaA proteins produces antibodies increase in serum, interleukin-17 (IL-17) release from splenocytes and protection against lethal *S. pneumoniae* challenge (Lu et al., 2015). Ply is other protein well conserved in *S. pneumoniae* and studies have shown that a non-toxic Ply-based vaccines stimulate antibodies production and protective effects in mice against *S. pneumoniae* lethal infection (Alexander et al., 1994; Petukhova et al., 2020; Thanawastien et al., 2021). Pneumoccocal histidine triad proteins (Pht) are a family of proteins that are present in the surface of *S. pneumoniae*, several of these proteins can induce production of protective antibodies against *S. pneumoniae* challenge in mice (Adamou et al., 2001). Moreover, several clinical trials are based in vaccines formulated with these antigens alone or in combination with others (Zhang et al., 2001; Bologa et al., 2012; Entwisle et al., 2017; Odutola et al., 2017).

Another alternative for protein-based vaccines is the generation of chimeric proteins, where antigens are designed *in silico* with multiple domains from *S. pneumoniae* proteins. Following this strategy, Suvorov et al., (2015) created a vaccine based on a chimeric protein composed of epitopes from PsaA, PspA, Spr1875, and FliC, called PSPF (Alexander and Ilya, 2015). Furthermore, recently, these authors tested this vaccine supplemented with a cell wall from *Lactobacillus rhamnosus* in mice. In this vaccine schedule, animals were immunized ay days 0, 14, and 28. At day 33 after immunization, serum and bronchoalveolar lavages (BALs) were recovered, and mice were challenged nasally with 10^7^ colony forming units (CFU) of *S. pneumoniae*. Two days later, quantification of antibodies in the serum and BALs showed that vaccination with PSPF+ cell wall from *L. rhamnosus* induced more IgG, IgA, and IgM than mice immunized with PSPF alone. Furthermore, lung bacterial counts in hemocultures showed that mice vaccinated with PSPF+ cell wall from *Lactobacillus rhamnosus* had lower lung CFU of *S. pneumoniae* than the other groups (Laiño et al., 2018).

Several adjuvants are used in vaccine formulation to increase the immune response or improve the vaccine’s long-term protection. Commonly, alum (Al(OH)_3_) or bacterial components like flagellins, toxoid components, or lipopeptides are used in vaccine formulations (Ogunniyi et al., 2007). New adjuvants that are in a research stage include the use of outer-membrane vesicles (OMV). OMV is obtained from membranes of Gram-negative bacteria and harbor various molecules like membrane proteins, LPS, peptidoglycan, periplasm, and cytoplasmatic proteins. These elements make OMVs potentially immunogenic, and an excellent adjuvant due to the multiple pathoen-associated molecular patterns (PAMPs) carried by them [for review, see Van Der Pol L. et al. 2015 (van der Pol et al., 2015)]. Malekan *et al.* compared the protective effect of recombinant PhtD vaccine combined with alum or OMVs as adjuvants. The OMVs used in this study were obtained from meningococcal cells (*Neisseria meningitidis* serogroup B) and characterized by electron microscopy and SDS-PAGE. The protective effect of immunization was evaluated with an intraperitoneal challenge of 1.4 x 10^7^ CFU of *S. pneumoniae* at day 46 after immunization in mice. IgG levels in blood showed a significant increase at day 35 in all immunized groups. However, the bacterial killing assay showed that the vaccine formulated with alum adjuvant was more bactericidal than with OMVs. In addition, 10 days after the challenge, mice immunized with proteins + Alum obtained a survival of 60%, while the mice immunized with proteins + OMVs only had a 30% survival (Malekan et al., 2020). Nevertheless, OMVs appear to be a new immunogenic option for future investigation and optimization.

#### Clinical trials

In a study of Bologa et al., (2012) that aimed to assess the safety and immunogenicity of 2 vaccine candidates for *S. pneumoniae*, a monovalent vaccine with choline-binding protein A (PcpA) and a bivalent PcpA-PhtD vaccine. Participants were vaccinated at day 0 and 30 and a follow-up of 30 day after immunization, where antibodies were evaluated at day 0, 30 and 60. The results showed that the concentration of antibodies against PcpA and PhtD increased after the second immunization, additionally there were no difference in the concentration of antibodies between the vaccine with adjuvant and the unadjuvanted one (Bologa et al., 2012).

In other study, Odutola et al. (2016) evaluated in a phase 2 clinical trial in infants a protein-based combined vaccine for *S. pneumoniae* and *Haemophilus influenzae*, where both pneumolysin and PhtD were included in a 10 serotype-specific polysaccharide conjugates of the pneumococcal and non-typeable *H. influenzae* protein D conjugate vaccine to obtain a combined one. This formulation was previously tested in adults and children, showing to be safe (Leroux-Roels et al., 2014; Odutola et al., 2016). In this study, 8-10 weeks old infants with nasopharyngeal carriage (NPC) of *S. pneumoniae* were immunized at 2, 3, and 4 months of age, while another group was immunized at 2, 4, and 9 months of age. The post-immunization visits were 1, 5, and 9 months after the third dose to obtain samples from the infants in both groups. The results showed that this vaccine formulation did not affect pneumococcal colonization in the NPC in any immunized group compared to the placebo group at the evaluated times. However, a significant increase in antibodies titers against Ply and PhtD in the vaccinated groups was observed compared to the control group, even when the infants had antibodies against *S. pneumoniae* in the preimmune sera. This is an important result because even if there is not an effect in the carriage, antibodies could protect from invasive infection (Odutola et al., 2017).

Entwisle et al. (2017) also performed a clinical trial where a vaccine called PnuBioVax (PBV) was evaluated. This vaccine was created from purified proteins of a *S. pneumoniae* (strain TIGR4) genetically modified to produce a non-toxic Ply (Ply6D). Therefore this vaccine contains multiple proteins like PspA, PlyD6, RrgA, heat shock proteins (Hsp) 60 among others (Hill et al., 2018). In the clinical trial, participants were immunized three times 28 days apart where safety and immunogenicity were evaluated. The results showed that the vaccine was safe even when in the higher doses (500 µg) the adverse events like headache or pain in the injection site increased. Also, the result showed that after immunization IgG titre increased in serum and antibodies against *S. pneumoniae* proteins like PspA or Ply had 2-fold increase in immunized participants. Moreover, purified Ply antibodies from participant showed an inhibition of hemolytic activity, which is an indicative of the neutralization capacity (Entwisle et al., 2017).

Although safety and immunogenicity in these clinical trials were positive, the protective effect against *S. pneumoniae* remains unknown. Also, the studies only evaluated Ig in serum and specific antibodies but a more complete study about cellular immunity in the vaccinated participants could help to understand in more detail the mechanism of protection of vaccines.

### Inactivated whole cell-based candidate vaccines

#### Pre-clinical studies

Vaccines based on inactivated whole cells have the advantage of carrying several types of antigens common to all serotypes of *S. pneumoniae* and are safe because the microorganism is completely inactivated (Jwa et al., 2018). In a study of Xu et al. (2014) designed an inactivated whole cell-based vaccine from a D39 *S. pneumoniae* called SPY1, which does not have capsule. The inactivation was carried out with ethanol 70%, and mice were immunized intranasally with 2x10^8^ CFU of the inactivated bacterium with cholera toxin as adjuvant. The animals were immunized four times at 1-week intervals, the control groups only received the adjuvant. Immunized mice were challenged with several strains of *S. pneumoniae* to evaluate survival and the results showed that the vaccine elicited a protective effect similar to the generated with a control group immunized with the PPV23 (Xu et al., 2014). In addition, mice inoculated with serum or cells from the spleen of immunized mice had a better survival against *S. pneumoniae* D39 challenge. These results are very promising considering that the vaccine is very protective in all the serotypes evaluated.

#### Clinical trials

Keech et al. (2020) evaluated the safety and immunogenicity of a *S. pneumoniae* whole-cell vaccine (wSp) in adults (NCT01537185) (Keech et al., 2020). In this study, 42 participants were randomized in 3 cohorts to receive 0.1, 0.3, or 0.6 mg of wSp or saline solution intramuscularly. Three doses were given to participants at 4-week intervals, and monitored at day 7 after inoculation "while adverse events through day 84. The group who received wSp well tolerated the vaccine formulation with reactivity in the first dose and pain at the injection site. Furthermore, the group who received wSp, elicited IgG rise in the serum of each immunized participant post-vaccination. In addition, IgG from vaccinated participants was more specific to proteins from *S. pneumoniae* like PspA or Ply. Remarkable, an increase in CD4^+^ T cells was seen in peripheral blood mononuclear cells (PBMCs) from the participants inoculated with 0.6 mg of this vaccine formulation. In addition, an increase in IL-17, CD40L, IL-12, and TNF-α was also observed. These data suggest that despite the small sample size, this vaccine is a promising candidate, and currently, it is in a phase 2 clinical trial stage in toddlers to assess dose escalation to 1 mg (Keech et al., 2020).

### Live attenuated candidate vaccines

#### Pre-clinical studies

This strategy remains an excellent option to obtain vaccines due to their low cost and good efficacy, however, attenuation should be effective and permanent to prevent virulence. Amonov et al. (2020) designed a vaccine with a serotype of *S. pneumoniae* mutant lacking the genes for capsule synthesis (*cpsE)* and endonuclease A (*endA)*. The *CpsE* is a gene related to capsule synthesis, and the mutation had been associated with an increase in the host immune response, lack of capsule, and lower virulence. Likewise, the *endA* gene is strongly associated with *S. pneumoniae* transformation capacity, so the lack of this gene could reduce the possibility of reintegration of *cpsE* and capsular formation (Amonov et al., 2020). The double *knockout* was made by homologous recombination in the strain D39 serotype 2 of *S. pneumoniae*, and capsular presence was assessed by transmission electron microscopy. The *in vivo* assays were carried out in mice, and intranasal immunization was performed twice with two weeks apart between the inoculations. The survival curve showed that the mouse group immunized with the double mutant-based vaccine had a 56% survival after the intranasal challenge with *S. pneumoniae*. Otherwise, the mouse group, challenged with the wild-type bacteria, showed a 50% survival. Besides the similarity in the survival percentage between the two groups, the above-mentioned investigators observed that the virulence of the double mutant bacteria was 23-fold lower than the wild-type *S. pneumoniae*. The authors also evaluated the IgG and IgM production from the sera of mice. They observed that antibodies against D39 serotype bacteria were increased in the sera of the mice immunized with the double mutant vaccine compared to those inoculated with the wild-type bacteria.

Jang et al. (2019) developed a live attenuated vaccine with a strain TIGR4 *lgt-mutant* of *S. pneumoniae. Lgt* gene codifies the Lipoprotein diacylglyceryl transferase (Lgt) (Jang et al., 2019), which participates in the production of lipoproteins in the bacterium. Lipoproteins are essential in several physiological roles in bacteria, for example, cell division, conjugation, adhesion, and immune evasion. *Lgt*-mutant *S. pneumoniae* has been shown to impair virulence, while immunogenic antigens are still present in the bacteria. Thus, the above-mentioned authors evaluated the protective capacity of this *S. pneumoniae* strain in mice. Survival assay showed this bacterium could colonize the mouse respiratory tract without death 10 days after inoculation, where CFU decreased at 72 h post-infection. Immunization was performed, intranasally, twice at 14-day intervals with TIGR4Δ*lgt*. A blood sample showed increased titers of IgG, IgM, and IgA, and the antibodies presented cross-reactivity with other strains of *S. pneumoniae.* A challenge to vaccinated mice was performed 10 days after the second immunization with the *S. pneumoniae* strains D39 and Wu2. Challenge with D39 strain in unimmunized mice generated 0% survival at day 7 post-infection while immunized mice had 75% survival at day 10 post-infection. Correspondingly, all control mice (not immunized) challenged with Wu2 strain died at day 7 after infection, while immunized mice had a 100% survival at day 10 after infection. Additionally, CFUs in the blood, lung, and nasopharynx were reduced in immunized mice.

Ramos-Sevillano et al. (2021b) evaluated the protective effect of colonization of 2 mutant strains of *S. pneumoniae* in mice. Both strains were generated from *S. pneumoniae* serotype 6B and contain mutations in locus *cps* (that encodes the capsule) and the second mutation was one of the virulence factors PspA and ProABC. Intranasal colonization was performed at day 0 and 14, while IgG in serum was evaluated at day 28 after colonization. Results showed that mice colonized with the mutant *S. pneumoniae* had increased level of IgG against *S. pneumoniae* antigens but compared with the wild type strain the effect was lower. Also, colonized mice with mutant strains had protection against septicemia challenge but they could not prevent recolonization with the wild type strain (Ramos-Sevillano et al., 2021a; Ramos-Sevillano et al., 2021b).

As reviewed, live attenuated vaccines are a good alternative to the develop of universal vaccines, however more research is required to assess the changes in the immune system when a participant is vaccinated. This is important because we could predict in a better way the protective effect of a potential vaccine. Usually, antibodies titers are used as indicative of the humoral immune response, but the effect of the vaccine in the cellular immune response should not be underestimated or omitted since it includes a large group of cells including CD4^+^ cells, a Th17 response and innate immune cells like, neutrophils, macrophages and dendritic cells (Nieto et al., 2013; Ramos-Sevillano et al., 2019).

### Pneumococcal proteins as carriers in conjugate vaccines

As discussed above, conjugate vaccines elicit a strong immune response and protection, but it is limited to the capsular serotypes included in the formulation, on the other hand, protein-based vaccines produce antibodies against a broad range of serotypes, but the protection still need to be assessed and confirmed. Considering this, some groups have studied the use of pneumococcal proteins as carries in the conjugate vaccines with the objective to have a strong immune response and protect against a broad range of serotypes (da Silva et al., 2017; Santiesteban-Lores et al., 2021). A vaccine candidate formulated by da Silva et al. (2017) that contain the protein PspA conjugated to the capsular polysaccharide serotype 14, immunized mice showed high IgG antibodies with opsonophagocytic activity but more importantly, the immunization protected from *S. pneumoniae* serotype 6. In other study Santiesteban-Lores et al. (2021) developed a vaccine where it was conjugated the protein PspA to the capsular polysaccharide serotype 6B. Immunization of mice with this formulation produced IgG antibodies against both the capsular polysaccharide and the protein PspA. Finally, Chichili et al. (2022) evaluated the safety, tolerability, and immunogenicity in humans of a vaccine called ASP3772, which was developed in a Multiple Antigen Presenting System (MAPS) platform. This system enables the creation of macromolecules with immunogenic characteristic like that of whole-cell vaccines by integrating several components from the pathogen (Zhang et al., 2013). ASP3772 was created with 24 biotinylated pneumococcal polysaccharides fused to 2 surface pneumococcal proteins (SP1500 and SP0785). The study was carried out in healthy adults aged 18 to 64 years old and in older adults aged 65 to 85 years old. The results showed that both age cohorts had limited reactions to the vaccine, including fatigue headache and myalgia. Vaccinated participants generated functional antibodies against the 24 serotypes included, more SP1500+SP0785-specific Th17 cell, and a higher IL-17 production in PBMCs post stimulation (Chichili et al., 2022). These approaches open another way to develop and improve the current vaccines in order to combine the advantages of both strategies and prevent serotype exchange.

### RNA as a platform for a *Streptococcus pneumoniae* vaccine

The COVID-19 pandemic has improved vaccine development globally, and currently, new strategies are available to create effective vaccines (Rauch et al., 2018; Mulligan et al., 2020). Specifically, RNA-based vaccines formulated against COVID-19 revealed a potential tool to generate immunity to *Streptococcus pneumoniae* infection. RNA is a molecule with particular and useful characteristics. It cannot integrate into the host genome, and it is a transient molecule that is degraded in a natural form in the host cell. The RNA design is relatively simple, and it allows the expression of antigens that mimics the original proteins (Mulligan et al., 2020). Several groups have worked in RNA vaccines before in treatments for cancer and other viruses like influenza (Kranz et al., 2016; Alberer et al., 2017; Feldman et al., 2019). Even so, vaccines against bacteria using this methodology have not been evaluated extensively. One study in 2004 evaluated the protective capacity of an RNA encoding MPT83 antigen during infection of *Mycobacterium tuberculosis* in mice (Xue et al., 2004). This RNA vaccine revealed short but important protection against a challenge, and it elicited a rise of antibody production in serum. Considering this study and the current advances in RNA-based vaccines, the idea of a vaccine with the main antigens from *S. pneumoniae* in this form is a promising alternative.

## Conclusions

Current vaccines for *S. pneumoniae* are effective but have some important problems related to their high cost, effectiveness depending on the individual’s age, and serotype replacement. Here we highlighted some examples of vaccine formulation strategies that attempt to surpass these problems from different perspectives. The use of inactivated whole-cell vaccines or protein-based vaccines could decrease production costs and serotype exchange, but in general, *in vivo* experiments using non-capsular vaccines could not protect entirely against bacterial challenge, maybe denoting the importance of capsule and its components as virulence factors. Additional experiments are needed to assess the protection in different *S. pneumoniae* serotypes.

Besides the difficulties in vaccine formulation to prevent *S. pneumoniae* disease, meaningful progress has been achieved, including the use of new adjuvants like OMVs, or new techniques for cell inactivation, such as gamma radiation, as well as new antigens for vaccine formulation and the use of pneumococcal proteins as carriers in conjugate vaccines. In addition, the pandemic context has also improved vaccine development, and new strategies need to be evaluated as potential alternatives to overcome current infectious diseases. Therefore, these discoveries, together with old vaccine formulations, could provide alternatives for more effective prophylactic solutions and save more lives, including immunocompromised patients, children, and the elderly.

## Author contributions

All authors listed have made a substantial, direct, and intellectual contribution to the work and approved it for publication.

## Funding

This study was supported by grants “Fondo Nacional de Ciencia y Tecnología de Chile” (FONDECYT) (# 1190830; 1190864; # 11706964), the Millennium Institute on Immunology and Immunotherapy, ANID - Millennium Science Initiative Program (ICN09_016/ICN 2021_045; ACE 210015 former P09/016-F) and the Regional Government of Antofagasta through the Innovation Fund for Competitiveness FIC-R 2017 (BIP Code: 30488811-0).

## Acknowledgments

We are thankful of Dr. Hernán F. Peñaloza (Pontificia Universidad Católica de Chile) for his revision of the manuscript and helpful comments.

## Conflict of interest

The authors declare that the research was conducted in the absence of any commercial or financial relationships that could be construed as a potential conflict of interest.

## Publisher’s note

All claims expressed in this article are solely those of the authors and do not necessarily represent those of their affiliated organizations, or those of the publisher, the editors and the reviewers. Any product that may be evaluated in this article, or claim that may be made by its manufacturer, is not guaranteed or endorsed by the publisher.
